# Comparative evaluation of community interventions for the immigrant population of Latin American origin at risk for Chagas disease in the city of Barcelona

**DOI:** 10.1371/journal.pone.0235466

**Published:** 2020-07-14

**Authors:** Jordi Gómez i Prat, Paula Peremiquel-Trillas, Isabel Claveria Guiu, Johanna Caro Mendivelso, Estefa Choque, Juan José de los Santos, Elena Sulleiro, Hakima Ouaarab Essadek, Pedro Albajar Viñas, Carlos Ascaso Terren

**Affiliations:** 1 Drassanes—Vall d’Hebron International Health Unit, International Health Programme, Institut Català de la Salut, Barcelona, Spain; 2 Asociación de Amigos de las Personas con Enfermedad de Chagas (Association of Friends of Chagas Affected Patients)—ASAPECHA, Barcelona, Spain; 3 Preventive Medicine and Epidemiology Department, Vall d’Hebron University Hospital, Barcelona, Spain; 4 Unit of Molecular Epidemiology and Genetics in Infections and Cancer, IDIBELL, Catalan Institute of Oncology, L'Hospitalet de Llobregat, Barcelona, Spain; 5 Fundación Mundo Sano–España, Madrid, Spain; 6 Department of Control of Neglected Tropical Diseases, Cluster for Communicable Diseases, World Health Organization, Geneva, Switzerland; 7 Department of Public Health, University of Barcelona, Spain, August Pi i Sunyer Biomedical Research Institute (IDIBAPS), Barcelona, Spain; Chinese Academy of Medical Sciences and Peking Union Medical College, CHINA

## Abstract

**Introduction:**

Chagas disease presents bio-psycho-social and cultural determinants for infected patients, their family members, close friends, and society. For this reason, diagnosis and treatment require an active approach and an integral focus, so that we can prevent the disease from creating stigma and exclusion, as is actively promoting access to diagnosis, medical attention and social integration

**Methodology:**

The study was conducted in the Metropolitan Area of Barcelona (Catalonia, Spain) from 2004 to 2017. After an increased detection rates of CHD in our region, the process of construction of community strategies started (2004–2013). Different community interventions with informational, educational, and communication components were designed, developed, implemented, and evaluated. The results of the evaluation helped to determine which intervention should be prioritized: 1) workshop; 2) community event; 3) in situ screening. Afterwards, those strategies were implemented (2014–2017).

**Results:**

Each of the three strategies resulted in a different level of coverage, or number of people reached. The *in situ* screening interventions reached the highest coverage (956 persons, 58.98%).Clear differences exist (*p-value*<0.001) between the three strategies regarding the percentage of screenings and diagnoses carried out. The largest number was in the *in situ* screening intervention, with a total of 830 persons screened despite the greatest number of diagnoses was among the workshop participants (33 persons, 20.75% of those screened). The prevalence of infection found is similar among the three strategies, ranging from 16.63% to 22.32% of the screened patients (*p-value* = 0.325).

**Conclusions:**

The results of the study show that community interventions seem to be necessary to improve access to diagnosis and treatment of CHD in the area of Barcelona. They also show which strategy is the most appropriate based on the detected needs of the community, the proposed objectives of the intervention, and the given socio-temporal context.

## Introduction

Chagas disease (CHD) is a disease caused by the parasite *Trypanosoma cruzi*. According to estimates by the World Health Organization (WHO), there are currently between 6 and 7 million infected people, predominantly in the continental territory of 21 Latin American countries [1]. As a consequence of population movement, mainly migration, a growing number of cases have been detected in recent decades in Canada and the United States of America, in 17 European countries, and in two in the West Pacific, characterizing a new epidemiological distribution worldwide [1,2]. In fact, CHD is, nowadays, a predominantly urban disease (two thirds of infected patients live in cities) and the means of non-vectorial transmission have acquired greater relevance [1,2]. It is estimated that in reality up to 75 million people in the world are at risk of infection [1].

In 2015 the WHO included CHD among the 21 Neglected Tropical Diseases and, like the others, one of the main challenges of its control is the detection of undiagnosed cases, estimating that worldwide, no more than 10% of infected patients have been diagnosed [1,3].

The biomedical, psycho-social, cultural, and anthropological characteristics of CHD are important determinants for those infected, their family members, and the society that surrounds them [4,5]. There are multiple complex barriers faced by migrant populations regarding access to CHD diagnosis and treatment. Psycho-social barriers, such as fear of the disease and stigma, are the most relevant. Other barriers are administrative, such difficulties accessing healthcare services [4–8]. An integral approach keeping these determinants in mind is essential for promoting access to diagnosis and treatment, along with social integration and prioritizing the elimination of the various personal and social barriers that characterize the disease [6–8].

In the recent years, approaches based on information, education, and communication have included the key analytical elements that are necessary to understand CHD and the problems that infected people face. These approaches have brought new perspectives that are both different and constructive to the families and close friends of patients, and to the community [7–9].

In addition, multiple decisive actions have recently been carried out by different stakeholders in the fields of public health, health systems, and the academic and research world, along with civil society (including those lead by different groups of people affected by CHD). These actions aimed to achieve better visibility, awareness, and promotion of access to diagnosis, treatment, and globally-applied research. One of the recent and most relevant initiatives was the creation of the International Federation of Associations of People Affected by Chagas Disease (FINDECHAGAS), in 2010 in Olinda (State of Pernambuco, Brazil), which today brings together more than 20 associations of affected people in the Americas, Europe, and the West Pacific.

In this context, the Public Health and Community team (eSPiC) of the Drassanes-Vall d’Hebron International Health Unit Drassanes-Vall d’Hebron (USIDVH), of the International Health Program of the Catalan Institute of Health—PROSICS, has worked in the community field since the year 2004 carrying out interventions that look to improve detection and access to diagnosis and treatment of CHD.

This article describes the experience of the first 13 years of this team’s work. First, the process of construction of the community strategies is explained, describing the design, development and implementation of the strategies. Afterwards, the resulting strategies are compared and evaluated.

## Methodology

The study is divided into two periods of time: the first one between 2004 and 2013, in which the community work process is built; and the second period from 2014 to 2017, in which three different community interventions about this population are implemented and evaluated.

### Area and period of the study

This work was conducted in the Metropolitan Area of Barcelona (Catalonia, Spain). In Catalonia in the year 2017, there were 7,555,830 inhabitants registered in the census, of which 13.78% were members of the immigrant population (1,041,362 inhabitants). Within the foreign population, 26.64% come from the Americas (277,435 inhabitants) [10]. Specifically, in 2017, the Bolivian population with residence permits in Catalonia was 30,655 people, of which 23,148 live in the province of Barcelona [10]. This shows a clear increase in the population of this group in our surroundings in the last decade; in 2007, 17,900 Bolivians lived in Catalonia, specifically 14,074 in the province of Barcelona. A total of, 9063 Bolivians were registered in the census within Barcelona’s city limits in 2017, which is less than those that lived there in 2007 (16,352) [11].

### Community intervention strategies

#### The process of construction of the community strategies (2004–2013)

From the year 2002–2004 the first cases of CHD in our surroundings were detected in a significant way, a disease which, until then, had not been detected in Catalonia [12].

The work began in 2004 with a clinical approach [13], followed by a socio-anthropological approach [5,14]. Once the situation was understood more deeply, an approach was begun from the public health field. This contributed to the creation, product of previous work, of the *Asociación de Amigos de las personas afectadas por la enfermedad de Chagas* (ASAPECHA, Association of Friends of Chagas affected Patients), which allowed for collaborative work to begin between primary care and specialized care. Subsequently a phase of integral approach started, incorporating the psycho-social aspects of the disease with clinical work. The cycle finished with a global approach proposal and with the definition of the best strategies to use, both in the improvement of access and in the management of clinical examination (Table 1).

**Table 1:** Stages of the construction and implementation process of the community strategies for improvement of access to diagnosis and treatment of Chagas disease in Catalonia: Approaches and objectives.

*2004–2013*					*2014–2017*
	Construction of Community Strategies				Implementation of the community strategies
2004–2005	2006–2007	2008–2009	2010–2011	2012–2013	
Clinical Approach	Socio-Anthropological Approach	Public Health Approach	Integral Approach	Global Approach	
• Establish specialized units • Establish diagnostic tests • Understand the characteristics of Chagas disease in a non-endemic area • Establish clinical protocols • Establish clinical pathways	• Understand the socio-anthropological characteristics of the disease • Begin community work	• Strengthen community work: creation of the Association of Friends of People Affected by Chagas Disease—ASAPECHA, Barcelona • Strengthen clinical pathways with Primary Care • Standardize the diagnostic tests in the public health system • - Train the Primary Care workers	• Strengthen community and social work • Preparation and editing of educational and promotional material • Establish work areas between care and the community: the “expert patient” program • Establish networks at the local and international level	Create a community network worldwide: PROSEVICHA Project • Prepare promotional material and publicity video	• Develop new strategies to increase access to diagnosis and treatment • Establish the characteristics, strategies, and implementers of the community intervention • Evaluate the strategies and interventions

#### Implementation of the strategies to improve the access to diagnosis and treatment of Chagas Disease: Community interventions (2014–2017)

The community interventions completed in this period have been organized into three groups: workshops, community events, and *in situ* screenings. The plan for these strategies was made by the community health team and integrated by a doctor, two nurses, and the community health agents (CHA). The CHA had also leaded the interventions accompanied on occasion by educators of community peers and/or multipliers. The whole community health team has been involved in all the interventions. CHA have been professionally trained as social mediators and also received specific training on community health. Community peers have been trained on Chagas disease by healthcare professionals within the community health team. Both, CHA and community peers, have played an important role in the implementation of the different strategies, by hosting workshops, informing in community events and facilitating *in situ* screening interventions.

These three proposed strategies are established according to:

1. The collective organization of the Latin American community, specifically Bolivians, living in Barcelona, which occurs mainly around leisure-cultural events.
2. The community health team observations regarding the strategies which had better acceptability among the Latin American community, specifically Bolivians, and that lead to an increased accessibility to the diagnosis and treatment of the affected people
3. The revised literature for the community approaches to tackle health problems, in which integrating an IEC approach promotes better results.

The workshops were organized thanks to the collaboration and involvement of different organizations and associations. The hosts of the workshops were the CHA. The group of participants was closed, with a maximum of 15 participants per workshop. The workshops lasted for one hour and they aimed to inform and educate. The material used was the result of work done during previous stages, such as that of the platform BeatChagas (www.beatchagas.info) [15].

In addition, there were the community events interventions, which involved CHA and peer educators. The objective was to get close to the population that is susceptible to contracting CHD by giving information about the disease at cultural events or crowded meetings (Table 2).

**Table 2:** Description of the different developmental phases of the implemented community strategies.

	Workshops	Community Events	*In Situ* Screening
**Recruitment and preparation**	Different organizations are contacted and meetings are set up to establish a network of contacts. Interested parties are contacted (in person or by phone) to attend the workshop.	The event is chosen and the organizers are contacted. The necessary logistics for the event are prepared: personal (healthcare professionals, CHA, community multipliers), along with educational and other materials.	
			A mobile unit for blood sampling is also brought to the event.
**Information and recruitment for the screening**	The workshop is conducted on the property of an association or at USIDVH. The general characteristics of Chagas disease and its impact at different levels is discussed, using materials from www.beatchagas.info. When the workshop is finished, the participants are offered the possibility of being screened for Chagas at USIDVH or other health centers in Barcelona.	An informative stand is set up at the event, at which the CHA and community multipliers inform others about the activity and Chagas disease.	
		People are informed about Chagas disease and the possibility of screening for Chagas at USIDVH or in other health centers in Barcelona.	People are informed about the disease and given surveys to assess their prior knowledge. Those that wish to be screened are accompanied to the mobile unit, where healthcare professionals conduct an interview.
**Scheduling and screening**	The professionals take note of the information of those who want to be screened, to contact them afterward. Visits are scheduled after contacting patients by phone. The visit to USIVDH occurs, following the normal protocol for Chagas disease screening. Blood samples are taken and processed in the laboratory. The patients are scheduled for follow-up visits to get their results.		Blood samples are taken and processed by the USIDVH lab. The patients are told that they will receive a phone call from USIDVH about the results or to schedule an appointment at the health center.
**Results and follow-Up**	The blood samples are processed in the microbiology lab at the Vall d’Hebron University Hospital. Follow-up is conducted according to the results obtained^1^: • When the test is negative, the patient is contacted by phone and informed. • When the test is uncertain (one test positive and the other negative) the patient is scheduled and a new blood test is performed. • If the test is positive, the patient is scheduled at USIDVH to undergo supplementary tests to determine the possibility of cardiac and/or digestive affectation, begin antiparasitic treatment, and follow-up.		

^1^The positive diagnosis is based on the consistency of two different and simultaneous techniques for the detection of anti-trypanosoma antibodies: one with a recombinant antigen (r-ELISA: Bioelisa Chagas Biokit, España) and another with an antigen lysate ORTHO *T*. *cruzi* ELISA, Johnson & Johnson, USA).

*CHA—Community Health Agents; USIDVH—International Health Unit Drassanes-Vall d’Hebron*.

Finally, the *in situ* screening interventions went a step further than community events by bringing both health information and screening closer to this susceptible population during their free time. This made easier for patients to take the test without having to travel far or go to the health center during their working hours.

### Microbiological testing and follow-up

The microbiological testing of the blood samples obtained during screening has been performed using one recombinant antigen EIA (CHAGAS ELISA IgG+IgM, Vircell, Spain). All of the samples with an index >0.9 were also tested simultaneously for one lysate antigen EIA (ORTHO *Trypanosoma cruzi* ELISA Test System, Johnson and Johnson, USA). Both techniques had to be concordant in with an index >0.9, to be considered a reactive serology. After the confirmation of a positive result, the CHD contacted the patients by phone or by person to attend the USIDVH. Access to antiparasitic treatment for CHD is universal in our healthcare setting, so patients were able to start their treatment just after their first clinical visit. They received cardiac and digestive tests in this first visits, to now the extension of CHD. Patients received medical and bio-psychosocial follow-up during the treatment and afterwards, first on a week basis and afterwards every 15 days.

### Statistical analysis

The analysis of the interventions was completed through Stata v14 (StataCorp. 2015. *Stata Statistical Software*: *Release 14*. College Station, TX: StataCorp LP). A univariate analysis was performed to describe the main characteristics of the participants in the different interventions. The interventions were compared to each other using the Pearson's chi-squared test, according to the number of the participants, the number of the screened persons and the result obtained in the screening test.

### Ethics statement

All participants gave oral consent to participate to the interventions and were actively enrolled to the community interventions once they received information about the activity. All patients who were screened gave oral consent to undergo the screening test as part of the health center’s routine screening protocol for CHD. Each patient’s consent was documented in their computerized medical history. The procedures performed during the screening are the ones recommended by the WHO. Data were analyzed after the completion of all the activities as a retrospective comparative analysis. Patients’ written consent was not possible to be obtained retrospectively because it was difficult to contact all of them. All patients’ data were codified and analyzed anonymously. No data containing personal or identifying information from the participants have been published. Vall d’Hebron Hospital Ethics Committee approved the study as a report of the results derived from regular clinical practice.

## Results

### Construction process of the community strategies (2004–2013)

#### 2004–2005: Clinical approach

Between 2004 and 2005 the first diagnosis of CHD in Barcelona were made, through a process of protocol, in the frame of a research project [13]. In this multi-focused study, an elevated percentage of participants were found to be infected with *T*. *cruzi* (41% of the total of participants in the study and up to 65% of the participants who were Bolivian) [13], demonstrating the existence of the disease in the area and its repercussions on public health.

This fact subsequently led to the publication of a document in consensus that was related to the diagnosis and treatment of imported CHD [16] in our surroundings in 2005. This document highlights the need to screen for *T*. *cruzi* in blood banks and in pregnant women. The document also shows the importance of working on awareness and training of health professionals to be relevant to carrying out the screening in health centers that specialize in tropical medicine. Likewise, in the following years, recommendations were published about possible cardiac and digestive effects [17,18].

#### 2006–2007: Socio-anthropological approach

After the clinical approach and its phases have been defined, the need to provide psyco-social support to these patients was detected [19] as a consequence of the daily assistance given to affected people and their family members. Between 2006 and 2008 a qualitative study was performed with the goal of understanding the meaning of CHD for Bolivian people in a migratory context [5]. From this study, several key themes were highlighted: the perception of inevitable death related to CHD, the fear of receiving the diagnosis of the disease, and, consequently, the limited willingness of patients to perform CHD diagnosis tests. The close link that is formed between death and CHD made it essential to question and revise how we establish contact between the patient and the health system, given that there is a confirmed lack of access from a social point of view.

#### 2008–2009: Approach from the fields of community health and public health

The formation of ASAPECHA in Barcelona in 2008 made it possible to unite carriers of the disease, family members, friends, and people with CHD. It guaranteed access to information about integral treatment in health and social services in a non-endemic context. At the same time, communication networks between the healthcare systems in the countries of residence and countries of origin were promoted. In addition, the existing communication and information networks throughout the world were reinforced by emerging new groups of affected people, such as the group in Barcelona, to promote a coordinated effort.

On February 24^th^, 2008, the publication of the news article “*Chagas*: *The Silent Disease*” in the newspaper *El Latino*, distributed in Spain, caused a rise in visits to USIDVH by people who come from areas where CHD is endemic, along with a rise in the number of diagnoses of CHD. That summer, another activity was held about the spread of the disease and the importance of screening during the Bolivian Heritage Festival, at the ASAPECHA/eSPiC stand. During this period, the Government of Catalonia began the compilation of the *“Protocol for screening and diagnosing Chagas disease in pregnant Latin American women and their newborns”* [20]. The Catalan Institute of Health (ICS), the leading healthcare service provider in Catalonia, also expedited requests for serology in the face of *T*. *cruzi* for all family doctors in the primary healthcare network. In addition, the clinical pathways were consolidated and screening protocols were stablished for people suspected of suffering from CHD [16,17,19–22].

#### 2010–2011: Integral approach

In this period, activities started being held during public community events and educational materials were produced in collaboration with institutions that specialize in the health field, predominantly eSPiC of USIDVH [23]. The work of previous stages was also consolidated by the participation of health professionals, eSPiC, and ASAPECHA in five celebrations put together by Latin American communities in the city of Barcelona. These three entities also monitored patients, not only at the clinical level but also at the psycho-social level.

In the year 2010, FINDECHAGAS (*International Federation of Associations of People Affected by Chagas Disease*) was created, a federation in which ASAPECHA participated regularly.

In Catalonia in 2011 the ICS [24] implemented the *“Expert Patient in Chagas Disease Program*” with the support of the WHO. The methodology that was followed in the sessions was established in the protocol of the *“Expert Patient Program*”[25], which has been used for other diseases and was adapted to the distinct features that CHD has. In this program, a patient with a diagnosis of CHD, trained with the eSPIC team, acts as an “expert patient” and trains and guides a group of newly affected patients. The goal of these sessions was for peers to inform and educate each other about CHD and to increase the knowledge and self-esteem of recently diagnosed participants. Furthermore, the sessions achieved greater participation and involvement of people in CHD awareness, either through ASAPECHA or through their participation in different community interventions.

#### 2012–2013: Global approach

In April of 2012, the project PROSEVICHA (*Project to Promote Awareness through Visualizing the Reality of People Affected by Chagas Disease*) was presented. Its goal was to make the public aware of the reality of people affected by CHD by showing the complex problems that affected people experience in different contexts with regard to access to diagnosis and treatment. To achieve this goal, different songs were prepared along with two publicity videos [15].

On April 14th 2013, the *“First Commemoration of the International Chagas Disease Day in Barcelona”* was held, together with ASAPECHA, FINDECHAGAS, and the MundoSano-España Foundation. At this event, which was held at the USIDVH, two parallel strategies were used: one for screening people who came from endemic countries, and another for promoting awareness and information for representatives of social, political, and healthcare entities. This way made it easier to promote access to diagnosis among the Latin American immigrant population, and to spread awareness of the importance of diagnosis and control at the individual, group, community, and institutional levels.

The event was publicized in places where socialization among the Latin American population is common (primary care centers, international phone booths, bars). Informative pamphlets, word of mouth, communication media (radio, websites), and invitations to representatives of those entities were used to spread word of the events. This was done with the support from healthcare personnel, administrative personnel, CHA, and members of ASAPECHA.

#### Process of implementing strategies to improve access to diagnosis and treatment of Chagas Disease: Community interventions (2014–2017)

The three community interventions that are described below were established as a result of the work done previously between 2004 and 2013 (Table 1). Between 2014 and 2017, 1,621 people in the city of Barcelona received intervention by the USIDVH, of which 1,101 (67.92%) underwent the diagnostic screening test for *T*. *cruzi*. Of all the people screened, 196 people (17.80%) have been diagnosed with CHD. More women than men participated in the different community strategies implemented. The majority of the participants (82.79%) were from Bolivian origin (Table 3). Between 2014 and 2017, 41 workshops were performed in community centers and health centers. In total there were 313 attendees, of whom 87.54% (274 of 313) requested an appointment to be visited at the USIDVH. A total of 58.03% of the patients (159 of 274) were screened, being CHD diagnosed in 33 people (20.75%) after two positive blood tests for *T*. *cruzi*. USIDVH and eSPiC also participated in 12 community awareness campaigns between 2014 and 2017: celebrations of Bolivian Mother’s Day, the Festival of the Alasitas, and the Consulate of Bolivia in Barcelona’s Open House Day. In total, 352 people were informed, of whom 77.84% (274 of 352) requested appointments at our unit afterward. A total of 112 of those people (40.88%) were screened, and a positive result (and consequently a diagnosis of CHD) was obtained in 25 people (22.32%). During this period, five efforts were made to screen for CHD *in situ* interventions: at the Bolivian Heritage Festival from 2014–2017 and at a concert for the Bolivian group *Los Kjarkas* in 2017. In total 956 people were reached and 830 of them (86.82%) were screened. CHD was confirmed in 138 patients (16.63%). The coverage, or number of people reached, is different in each of the three proposed strategies; the greatest coverage was observed in the *in situ* screening interventions (956 persons, 58.98%) (Table 3).

**Table 3:** Summary of the results obtained in the different community strategies implemented between 2014–2017.

Strategy (year)	Participants	Bolivians		Women		Visited at USIDVH		Screened for Chagas disease		Affected of Chagas disease	
**Workshops ^1^**	**Total**	***n***	***%***	***n***	***%***	***n***	***%***	***n***	***%***	***n***	***%***
9 workshops (2014)	70	47	67.14	57	81.43	54	77.14	37	68.52	7	18.92
7 workshops (2015)	42	25	59.52	23	54.76	34	80.95	28	82.35	7	25.00
9 workshops (2016)	68	41	60.29	49	72.06	61	89.71	29	47.54	3	10.34
16 workshops (2017)	133	119	89.47	83	62.41	125	93.98	65	52.00	16	24.62
***Global***	***313***	***232***	***74*.*12***	***212***	***67*.*73***	***274***	***87*.*54***	***159***	***58*.*03***	***33***	***20*.*75***
*Persons per intervention*	*7*.*63*	*5*.*66*		*5*.*17*		*6*.*68*		*3*.*88*		*0*.*80*	
**Community events ^2^**	**Total**	***n***	***%***	***n***	***%***	***n***	***%***	***n***	***%***	***n***	***%***
3 events (2014)	28	28	100.00	17	60.71	27	96.43	7	25.93	5	71.43
3 events (2015)	108	98	90.74	64	59.26	72	66.67	30	41.67	6	20.00
3 events (2016)	95	85	89.47	63	66.32	86	90.53	32	37.21	9	28.13
3 events (2017)	121	94	77.69	85	70.25	89	73.55	43	48.31	5	11.63
***Global***	***352***	***305***	***86*.*65***	***229***	***65*.*06***	***274***	***77*.*84***	***112***	***40*.*88***	***25***	***22*.*32***
*Persons per intervention*	*29*.*33*	*2*.*42*		*19*.*08*		*22*.*83*		*9*.*33*		*2*.*08*	
***In situ* screening ^3^**	**Total**	***n***	***%***	***n***	***%***	***n***	***%***	***n***	***%***	***n***	***%***
1 intervention (2014)	181	139	76.80			131	72.38	131	100.00	35	26.72
1 intervention (2015)	264	264	100.00	166	62.88	264	100.00	264	100.00	45	17.05
1 intervention (2016)	164	164	100.00	98	59.76	164	100.00	164	100.00	34	20.73
2 interventions (2017)	347	238	68.59	193	81.09	271	78.10	271	100.00	24	8.86
***Global***	***956***	***805***	***84*.*21***			***830***	***86*.*82***	***830***	***100*.*00***	***138***	***16*.*63***
*Persons per intervention*	*191*.*20*	*161*.*00*				*166*.*00*		*166*.*00*		*27*.*60*	

1.Workshops were performed in health centers and in community centers. In the year 2014, the participants were from 9 different countries. In 2015, the participants were from 6 different countries while in 2016 from 10 and in 2017 from 4 countries. Those countries were: Venezuela, Ecuador, Perú, Honduras, Nicaragua, El Salvador, Colombia, Argentina, Dominican Republic, Paraguay and Puerto Rico.

2.In 2014, the participants in the community events where only from Bolivia, while in 2015, 2016 and 2017 they came from 6 different countries: Ecuador, Perú, Colombia, Paraguay, México and Spain.

Data on patients visited at USIDVH and screened for Chagas disease are the same as the in situ screening intervention was performed in a mobile unit from USIDVH. For 2014 intervention, no data on gender are available. In 2015, interventions were only among people from Bolivian origin.

The results obtained show that the number of people who request an appointment after doing the workshop is higher than those who do so after community events (87.54% and 77.84%, respectively), despite the fact that no significant differences wereobserved (*p-value* = 0.309). Clear differences do exist between the three strategies in terms of the percentage of screening tests carried out (*p-value*<0.001). The largest number of patients screened occurred atthe *in situ* screening interventions; the lowest number occurred in the community events, with 112 people screened (40.88%). However, the greatest number of diagnoses was made among the participants in community events and workshops (25 and 33 persons respectively, 22.32% and 20.75% of those screened respectively). The prevalence of infection found is similar among the three strategies, ranging from 16.63% to 22.32% of the screened patients, with no significant differences (*p-value* = 0.325) (Table 3).

It is worth highlighting that there was a higher percentage of women participating in the different interventions conducted compared to men, ranging from 54.76% to 81.43, with no statistical differences regarding the type of intervention (*p-value* = 0.743) (Table 3).

## Discussion

The first years of work (2004–2013) fostered the establishment and reinforcement of the three types of community interventions thatwork was focused during subsequent stages. The increase in disease detection and parallel improvement in the quality of both individual and collective care, which were both results of the different approaches taken initially, were determinant factors in decidingon the community interventions to be carried out starting in 2014. The final goal was integral care for people affected by CHD by cultivating an improvement in their quality of life [7], as has been observed.

The results obtained in the three community interventions (workshops, community events, and *in situ* screenings) conducted starting in 2014 show differences between each other regarding the number of participants and the total of screenings performed, although those differences were not statistically significant. The participants that the interventions focused on were principally of Bolivian origin, since previous studies in Europe verify that there is a higher prevalence of CHD in this group [3,26].

We observed a larger percentage of women participating in all of the interventions conducted. Other publications had already shown that there was more participation among women than men, both in the awareness events and in their interest and need to perform the screening in relation to Chagas [24,27,28]. According to previous studies, women show more concern and interest in screening, mainly because of feelings of guilt, worry, and responsibility for the potential transmission of the disease to their children [5]. As congenital transmission of CHD is well known and women are conscious of it, this may lead to and increased participation of women in the interventions which is also crucial to control this way of transmission.

The potential number of people reached depends on the chosen strategy. The *in situ* screening interventions allow us to reach a greater number of people, but also require a greater effort in terms of people and organization, as can be observed in Table 2. Likewise, the community events allow more people to be accessed even though the event is limited to being informative-educational because of the lack of possibility of *in situ* diagnosis; with the idea of setting up an appointment later on. In reference to the workshops, the investment of time is higher and fewer people are reached. Nevertheless, the results obtained show that the percentage of people who request an appointment after doing the workshop is higher than those who do so after community events (87.54% vs 77.84%, respectively), even though no significant differences have been observed (p-value = 0.309). This suggests that the workshops, since they are an educational activity with fewer participants, allow for greater understanding of the disease and its current predicament and, at the same time, allow for a more detailed follow-through with scheduling appointments. At the community events, educational actions are more difficult, since we must speak to many more people over a smaller period of time, limiting it to an action that is merely informative without follow-up.

Regarding access barriers to screening, the interventions were performed were adapted to suit the community. They were done close to the homes and workplaces of the Latin American community during non-working regular hours, thus facilitating access of those who were interested. Relevant differences are made evident among the three community interventions in terms of completion of the screening. In the case of *in situ* screenings, the percentage of screened people was higher than that observed in the rest of the interventions. This is because the main objective of this type of interventions is to complete the screening in a specific population at the time of intervention, with the overall goal of improving follow-up and adherence of the patients to their integral treatment. When the patients undergo the diagnostic test in the intervention, they are closely linked to their follow-up treatment. Once the screening test had been performed, the results were given by phone call, minimizing the number of visits at the clinic. In case of positive result and need of treatment and follow-up visits, clinic schedules were very adaptable to patients. The patients with a positive result were given a medical appointment in our clinic, having been located and advised by the same community healthcare team that had intervened at the events. This helps form bonds of trust and cultural adaptation, which had already begun at the festival

In the different published studies, we observed difficulty with both recruitment and follow-through. In a study conducted in Italy, 1305 people were screened as a result of screening workshops. Of those screened, 223 people (17%) had Chagas, and there was a large number of patients lost in the follow-through [28].

In relation to follow-up and the benefit of the intervention on the part of the community health team, the study completed in Barcelona in the frame of the congenital CHD program showed that of the total number of newborns that should have been screened according to protocol, 42 (24%) were not screened. The team of CHA, through community interventions, recovered 30 of them, leaving only 7% of patients who still needed the screening recommended in the protocol [29]. Another study done in Madrid shows how out of 352 participants in relevant talks, 276 (78.4%) were tested immediately for *T*. *cruzi* [30].

Because of these facts, in our interventions we observed a higher percentage of people screened during the *in situ* screening, followed by the workshops (58.03%), and finally by the community events (40.88%). This suggests again that the educational piece behind the screenings and workshops is better than that of the community events, reinforcing the idea that the community events are limited to being simply informative actions. The fact that CHD it is a disease in which psycho-social aspects play such a relevant role means that it is very necessary to approach it from an educational point of view, to transform a collective conscience affected by stigmas brought from the past. Also, the higher number of persons screened in the *in situ* screening interventions shows that facilitating the access to screening tests, as performing them in cultural/social events and in non-working days, increases the accessibility to potentially CHD affected patients.

Finally, the prevalence of disease in the three types of interventions are similar (p-value = 0.325), although there are differences that deserve to be highlighted. We observed a prevalence of 22.32% (95% CI: 14.99–31.16%) in the community events; of 20.75% in the workshops (95% CI: 14.74–27.89%); and of 16.63% in the *in situ* screening (95% CI: 14.15–19.33%).

Previous studies conducted in Catalonia by the Catalonian Blood Bank showed a seroprevalence of *T*. *cruzi* infection of 10.2% in Bolivian donors [31]. In published studies, the prevalence of CHD observed among the Bolivian population living in Europe is 18.1% (95% CI: 13.9–22.7%), which differs from the prevalence of the rest of the Latin American population that lives in the territory in which is 4.2% (95% CI: 2.2–6.7%) [26]. In Spain, a prevalence of 27.7% has been described among Bolivian population [27]. The higher prevalence observed in our study, compared to the seroprevalence study among Bolivian donors, is attributable to the fact that our interventions were designed to reach at-risk patients, where self-considered patients at risk will be more likely to attend to the interventions and to the screening. Since there was little informative time and minimal educative action in community events, only those that seemed most at risk of being affected were reached. This means that there could be a selection bias in the intervention and that the other interventions could carry the same self-selection.

The collaborative work between team members facilitates the implementation of the above-mentioned strategies, complementing clinical and social aspects. CHA are responsible for establishing social networks and contacts; expert patients/peer educators are in charge of informing and educating the participants; public health nurses and doctor take care of the strategy and its implementation; and the clinical team handles the clinical aspects, including diagnoses, treatment and follow-up. We believe that the success of our interventions owes itself to the fact that eSPiC relies on a team of CHA that understand the particular features of the Latin American community, specifically the Bolivian community, and knows their social networks and meeting places. Therefore, they were able to get past some of the psycho-social barriers that impede the population that is susceptible to suffer from CHD from accessing necessary medical attention [32]. Additionally, the “expert patients” and the peer educators completed the effort by approaching the needs and perceptions of the population that is likely to suffer from CHD. All those involved in the planning and execution of the strategies played key roles, and without them it would not have been possible to achieve such successful results.

These interventions have been set within a more integral framework of information, education and communication which has been conducted since the very beginning of the present study in eSPiC, becoming education necessary to overcome these psycho-social barriers [33]. Programs like this have also been used successfully for other diseases such as HIV in adolescents and in other types of healthcare, such as primary care and mother/child healthcare [34–36]. This suggests that the presence of CHA improves the effectiveness of the community interventions.

As for the limitations of the study, it should be noted that we do not have information on the total number of attendees at the community events in which *in situ* screening interventions were performed. Data collection was planned after the project began, so some variables that could have been of great interest were not gathered, such as: age and sex, both in those visited at USIDVH but also among those screened or affected by CHD; number of pregnant women participating in the different interventions; country of origin; socioeconomic and demographic data; etc. This information is very difficult to collect retrospectively. In this sense, data were also not registered systematically and prospectively, causing missing information. If the data collection had been done in a way that made this information available, the study would be much more informative and the impact could be assessed in a more accurately. There is also some possible bias present in this study regarding missing information, as the populations in the different interventions are assumed to be comparable and they may not be. Considering that the majority of the participants were from Bolivia, the results obtained may be interpreted cautiously when extrapolating to other endemic countries, as socio-demographic characteristics may be different.

In our context, the migratory experience transforms the perception that people have of themselves and of CHD [19]. It is important to prevent people from being stigmatized once they contract the disease, since this can reinforce the process of social exclusion. For this reason community work becomes very important, as it helps reverse these perceptions and social exclusion. Active and organized participation of affected patients contributes significantly in the prevention and awareness of the disease.

The characterization of the different community interventions available to increase detection of cases of CHD, based on the context and the reality of the different populations, is an opportunity to optimize the different screening strategies. It is necessary to adjust resources and improve efficiency in order to increase the number of patients diagnosed and improve the follow-up care of those affected.

The choice of the strategy should consider different aspects, such as the possibilities in terms of resources and knowledge of the teams involved, the available social network, the presence of civil society organizations, the barriers on access to healthcare for those affected regarding their administrative situation, etc. Nevertheless, our results suggest that when the prevalence of CHD is unknown in the targeted groups, the community event strategy should be prioritized because it allows reaching a large and diverse audience, to access equal or better prevalence of disease, and it requires fewer people and materials for the intervention, which should be less expensive and more effective. When the prevalence of CHD in a certain population is known to be high, the most adequate strategy is the *in situ* screening, along with the workshops using CHA, peer educators, community leaders, and associations. In our opinion, it would be advisable to conduct cost-efficiency studies to better understand, and be able to exactly quantify, the cost of these interventions related to their impact in accordance with the prevalence of disease in a specific environment.

## Conclusion

The community intervention strategies in different non-endemic contexts should be adapted, both in their preparation and their execution, to the characteristics of each context.

An intervention based on the community that involves community health teams, including health professionals, CHAs and peer educators can be more effective than the habitual routine of health centers. This is because of the psycho-emotional and socio-anthropological characteristics of CHD, and because of the fact that the community health teams, CHA and peer educators are experts in this approach and have access to resources and strategies that are adequate in this situation.

These approaches allow for bonds of mutual trust between professionals and the community that could be helpful in the future development of health promotion strategies.

## Abbreviations

ASAPECHA: Asociación de Amigos de las personas afectadas por la enfermedad de Chagas—Association of Friends of Chagas Affected Patients
CHA: Community Health Agents
CHD: Chagas Disease
CI: *Confidence Interval*
eSPiC: Public Health and Community Team
ICS: Catalan Institute of Health
USIDVH: International Health Unit Drassanes -Vall d’Hebron
WHO: World Health Organization
